# Porcine Coronavirus HKU15 Detected in 9 US States, 2014

**DOI:** 10.3201/eid2009.140756

**Published:** 2014-09

**Authors:** Leyi Wang, Beverly Byrum, Yan Zhang

**Affiliations:** Ohio Department of Agriculture, Reynoldsburg, Ohio, USA

**Keywords:** porcine coronavirus, HKU15, United States, deltacoronavirus, Deltacoronavirus genus, Coronaviridae, diarrheal disease, outbreaks, pigs, viruses, Midwest, porcine epidemic diarrhea virus, PorCoV

**To the Editor:** Porcine coronavirus (PorCoV) HKU15 is a single-stranded, positive-sense, enveloped RNA virus belonging to the genus *Deltacoronavirus* (family *Coronaviridae*). PorCoV HKU15 was first identified in 2012 in a surveillance study from China (*1*). Until February 2014, however, the role of this virus strain in clinical diseases of pigs had not been reported. 

We recently reported the detection of PorCoV strain HKU15-OH1987 in feces samples from sows and intestine samples from piglets in Ohio, United States; the infected animals were from swine farms where outbreaks of diarrheal disease had occurred in late January and early February 2014 (*2*). Genetic analysis showed that HKU15-OH1987 is closely related to 2 deltacoronavirus strains that were detected in Hong Kong, China, in 2012: HKU15-155 and HKU15-44 (*2*). We also demonstrated the presence of histopathologic lesions in the small intestines of PorCoV HKU15–infected piglets with diarrhea (L. Wang, unpub. data). In April 2014, a novel swine enteric coronavirus disease caused by PorCoV HKU15 or porcine epidemic diarrhea virus was reported to the World Animal Health Organization by the US Department of Agriculture (http://www.oie.int/wahis_2/public/wahid.php/Reviewreport/Review?page_refer=MapFullEventReport&reportid=15133).

PorCoV HKU15 is now recognized as a key pathogenic cause of diarrheal diseases in pigs in the United States. However, the geographic distribution and genotype diversity of PorCoV HKU15 in this country are still not clear. To further our knowledge of the virus, we analyzed swine samples that had been submitted for diagnosis of diarrheal disease from farms in 10 US states. We report the detection and phylogenetic analyses of PorCoV HKU15 strains from these samples.

Between February 7, 2014, when PorCoV HKU15-OH1987 was first identified in Ohio (*2*), and April 9, 2014, the Animal Disease Diagnostic Laboratory of the Ohio Department of Agriculture received >2,000 swine samples from farms in 10 US states for diagnosis of diarrheal disease. The states from which samples had been submitted were Minnesota, South Dakota, Nebraska, Illinois, Indiana, Michigan, Kentucky, Pennsylvania, Maryland, and Ohio. Among those samples, 435 were selected to be tested for the presence of PorCoV HKU15. A real-time reverse transcription PCR assay targeting the membrane protein gene was used to identify PorCoV HKU15. Samples with a cycle threshold value of <35 were considered positive on the basis of validation data using the cloned membrane protein gene (data not shown). Of the 435 samples, 109 (25%) from 9 states (all states mentioned above, excluding Maryland) were positive for PorCoV HKU15 by real-time reverse transcription PCR (Figure). Of those 109 samples, 19 (17%) were also positive for porcine epidemic diarrhea virus. This result suggests that PorCoV HKU15 is prevalent among pig populations in the major pig-producing US states.

**Figure:**
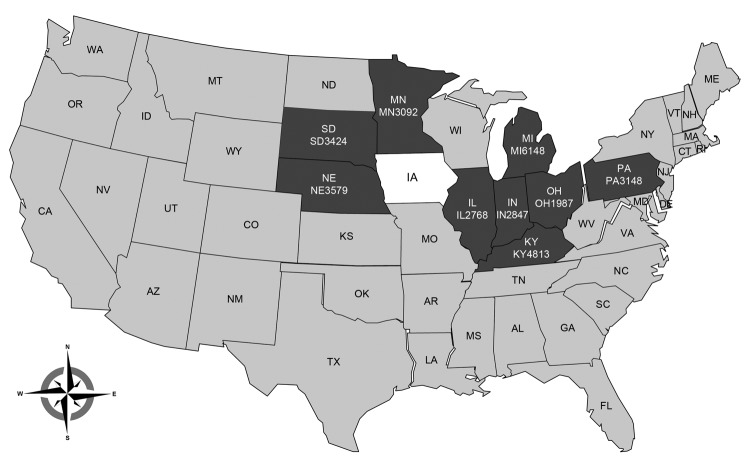
US states with swine samples positive for porcine coronavirus (PorCoV) HKU15, between February and April 2014. A total of 435 samples from 10 states were selected to be tested for the presence of PorCoV. Of those samples, 109 (25%) from 9 states (dark gray) were positive for PorCoV HKU15. Another recent article reported the presence of PorCoV HKU15 in Iowa (white) (*3*). Strain names are indicated below state abbreviations.

To determine the genetic diversity of PorCoV HKU15 strains from the 9 states, we conducted whole-genome sequencing for 1 strain from each state by using 16 pairs of previously described, overlapping primers (*2*). Strain names were designated by the state abbreviation and case number. The complete sequence for HKU15-OH1987 was reported previously (*2*). Sequence analysis showed that strains from South Dakota (SD3424), Nebraska (NE3579), Illinois (IL2768), Indiana (IN2847), Kentucky (KY4813), Michigan (MI6148), and Pennsylvania (PA3148) have the same genome size (25,422 nt) as OH1987, and whole-genome pairwise comparison showed that they share a high nucleotide similarity (>99.8%). Furthermore, all of the isolates share high nucleotide similarity (98.9%–99.2%) with the 2 PorCoV HKU15 strains in GenBank, HKU15-155 and HKU15-44. 

A phylogenetic tree constructed by using the entire sequence showed that all PorCoV HKU15 strains from the United States clustered together in 1 clade of the genus *Deltacoronavirus* with HKU15-155 and HKU15-44 (Technical Appendix Figure, panel A). This finding indicates that 1 genotype of PorCoV HKU15 is currently circulating in multiple US states. This result was further supported by phylogenetic trees constructed by using the full-length amino acids of spike and nucleocapsid proteins (Technical Appendix Figure, panels B, C). Because of limitation of the samples received, only a partial genome sequence was determined for strain MN3092 from Minnesota. However, on the basis of the spike and nucleocapsid protein sequence analyses, it is highly likely that the entire genome of the Minnesota strain is genetically identical to that of the other 8 strains (Technical Appendix Figure, panels B, C).

In addition to the 9 states reported in this study, Iowa has also had a recent detection of PorCoV HKU15 (*3*). Thus, PorCoV HKU15 has been detected in 10 of the 50 US states, and those 10 states mainly cluster in the midwestern United States (Figure). As with data collected for porcine epidemic diarrhea outbreaks by the US National Animal Health Laboratory Network (http://www.nahln.org/default/), data collected on the geographic location and numbers of PorCoV HKU15 cases is also required to be reported weekly. 

Earlier reports support avian coronaviruses as the gene source for *Deltacoronavirus* spp. (*1**,**4*). To confirm this, surveillance for PorCoV should be carried out among birds. Moreover, effective control strategies, including vaccine development, should be in place for prevention and control of infections caused by PorCoV HKU15.

Technical Appendix Phylogenetic tree of 4 genera of coronaviruses (*Alphacoronavirus*, *Betacoronavirus*, *Gammacoronavirus*, and *Deltacoronavirus* spp.), including 9 US strains of porcine coronavirus HKU15.

## References

[1] Woo PC, Lau SK, Lam CS, Lau CC, Tsang AK, Lau JH, Discovery of seven novel mammalian and avian coronaviruses in the genus *Deltacoronavirus* supports bat coronaviruses as the gene source of *Alphacoronavirus* and *Betacoronavirus* and avian coronaviruses as the gene source of *Gammacoronavirus* and *Deltacoronavirus.* J Virol. 2012;86:3995–4008 . 10.1128/JVI.06540-11 22278237 PMC3302495

[2] Wang L, Byrum B, Zhang Y. Detection and genetic characterization of a deltacoronavirus in pigs in the United States. Emerg Infect Dis. 2014. 10.3201/eid2007.140296 PMC4073853 24964136

[3] Li G, Chen Q, Harmon KM, Yoon KJ, Schwartz KJ, Hoogland MJ, Full-length genome sequence of porcine deltacoronavirus strain USA/IA/2014/8734. Genome Announc. 2014;2:e00278-14. 10.1128/genomeA.00278-14 PMC3983307 24723718

[4] Chu DK, Leung CY, Gilbert M, Joyner PH, Ng EM, Tse TM, Avian coronavirus in wild aquatic birds. J Virol. 2011;85:12815–20 . 10.1128/JVI.05838-11 21957308 PMC3209365

