# Supplementary material for: Porcine Coronavirus HKU15 Detected in 9 US States, 2014
Source: Emerg Infect Dis. 2014 Sep;20(9):1594–5. doi: 10.3201/eid2009.140756 (PMC4178395; doi:10.3201/eid2009.140756)
Supplement: Technical Appendix — Phylogenetic tree of 4 genera of coronaviruses (Alphacoronavirus, Betacoronavirus, Gammacoronavirus, and Deltacoronavirus spp.), including 9 US strains of porcine coronavirus HKU15. [file 14-0756-Techapp-s1.pdf]

Article DOI: http://dx.doi.org/10.3201/eid2009.140756

Porcine Coronavirus HKU15 Detected in 9 US States, 2014

Technical Appendix

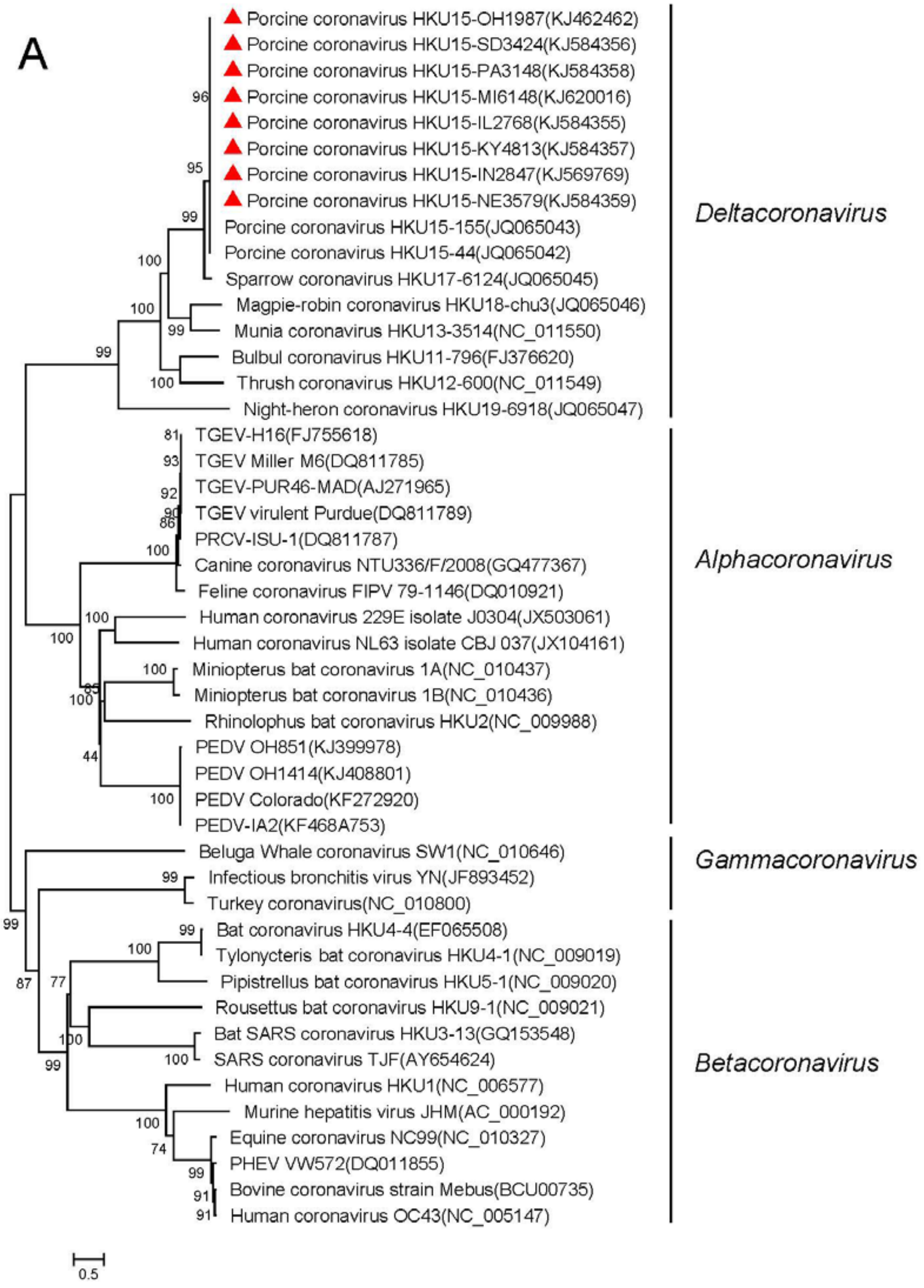

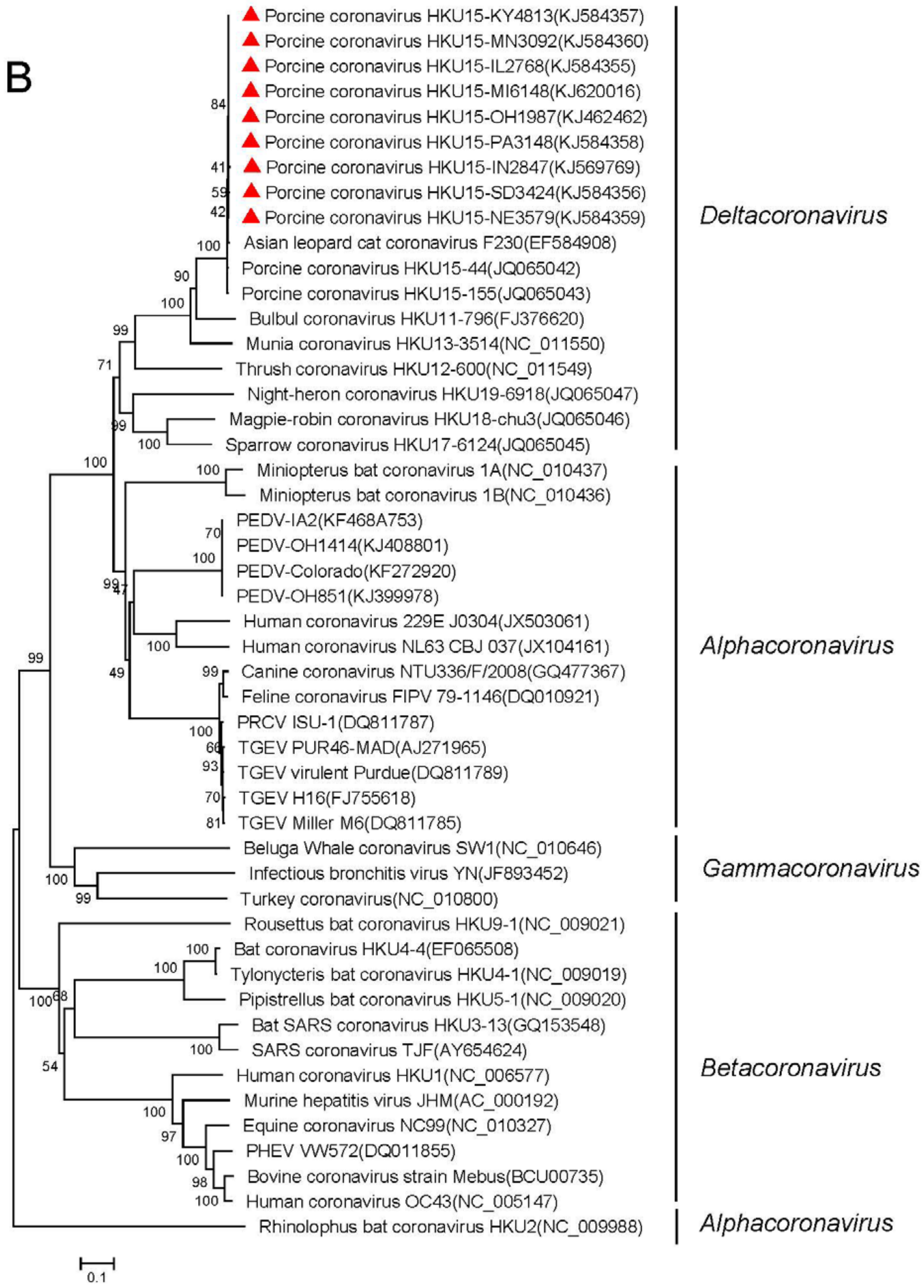
B
Porcine coronavirus HKU15-KY4813(KJ584357)
Porcine coronavirus HKU15-MN3092(KJ584360)
Porcine coronavirus HKU15-IL2768(KJ584355)
Porcine coronavirus HKU15-MI6148(KJ620016)
Porcine coronavirus HKU15-OH1987(KJ462462)
Porcine coronavirus HKU15-PA3148(KJ584358)
Porcine coronavirus HKU15-IN2847(KJ569769)
Porcine coronavirus HKU15-SD3424(KJ584356)
Porcine coronavirus HKU15-NE3579(KJ584359)
Asian leopard cat coronavirus F230(EF584908)
Porcine coronavirus HKU15-44(JQ065042)
Porcine coronavirus HKU15-155(JQ065043)
Bulbul coronavirus HKU11-796(FJ376620)
Munia coronavirus HKU13-3514(NC_011550)
Thrush coronavirus HKU12-600(NC_011549)
Night-heron coronavirus HKU19-6918(JQ065047)
Magpie-robin coronavirus HKU18-chu3(JQ065046)
Sparrow coronavirus HKU17-6124(JQ065045)
Miniopterus bat coronavirus 1A(NC_010437)
Miniopterus bat coronavirus 1B(NC_010436)
PEDV-IA2(KF468A753)
PEDV-OH1414(KJ408801)
PEDV-Colorado(KF272920)
PEDV-OH851(KJ399978)
Human coronavirus 229E J0304(JX503061)
Human coronavirus NL63 CBJ 037(JX104161)
Canine coronavirus NTU336/F/2008(GQ477367)
Feline coronavirus FIPV 79-1146(DQ010921)
PRCV ISU-1(DQ811787)
TGEV PUR46-MAD(AJ271965)
TGEV virulent Purdue(DQ811789)
TGEV H16(FJ755618)
TGEV Miller M6(DQ811785)
Beluga Whale coronavirus SW1(NC_010646)
Infectious bronchitis virus YN(JF893452)
Turkey coronavirus(NC_010800)
Rousettus bat coronavirus HKU9-1(NC_009021)
Bat coronavirus HKU4-4(EF065508)
Tylonycteris bat coronavirus HKU4-1(NC_009019)
Pipistrellus bat coronavirus HKU5-1(NC_009020)
Bat SARS coronavirus HKU3-13(GQ153548)
SARS coronavirus TJF(AY654624)
Human coronavirus HKU1(NC_006577)
Murine hepatitis virus JHM(AC_000192)
Equine coronavirus NC99(NC_010327)
PHEV VW572(DQ011855)
Bovine coronavirus strain Mebus(BCU00735)
Human coronavirus OC43(NC_005147)
Rhinolophus bat coronavirus HKU2(NC_009988)
Deltacoronavirus
Alphacoronavirus
Gammacoronavirus
Betacoronavirus
Alphacoronavirus
0.1

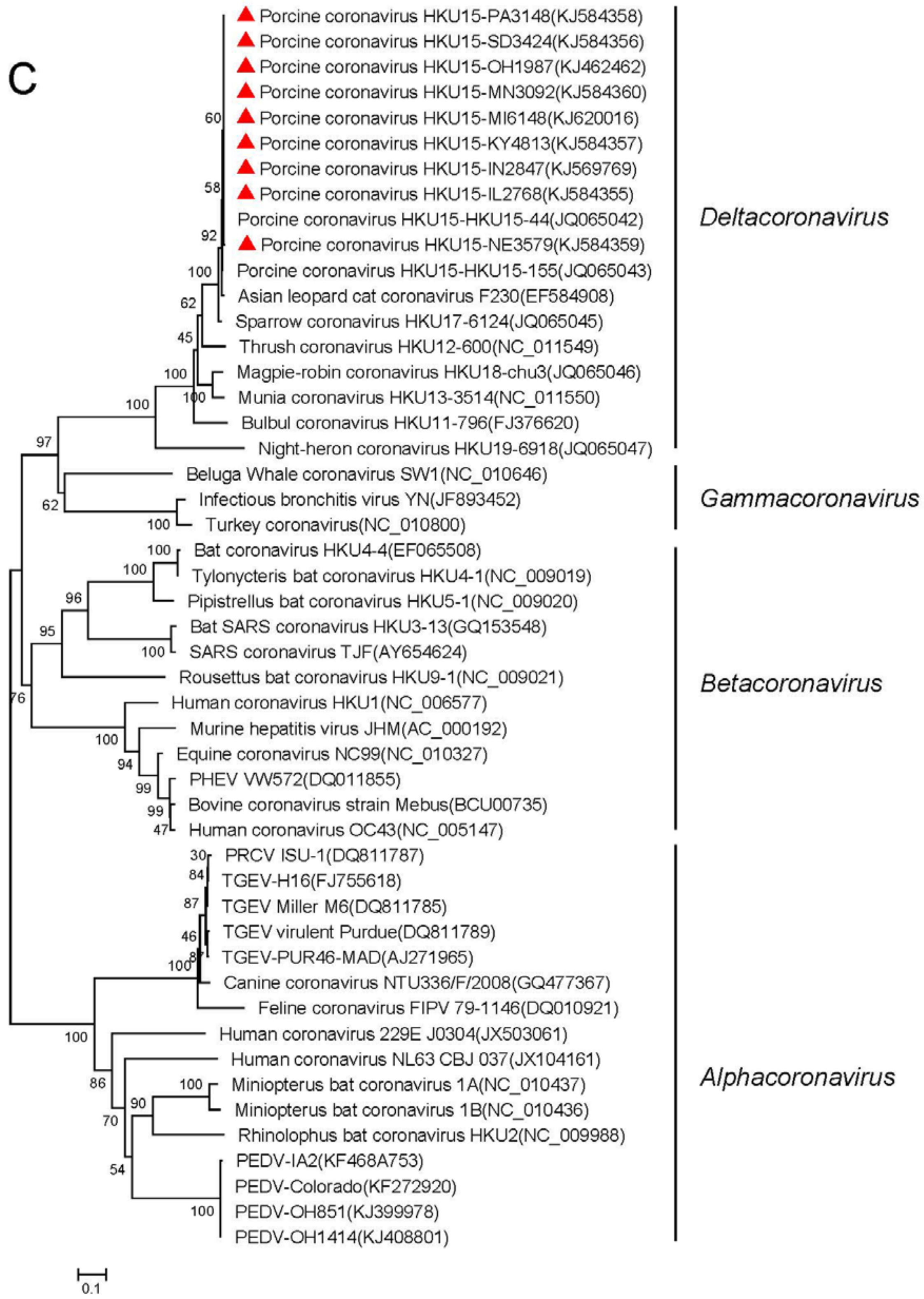

C
Porcine coronavirus HKU15-PA3148(KJ584358)
Porcine coronavirus HKU15-SD3424(KJ584356)
Porcine coronavirus HKU15-OH1987(KJ462462)
Porcine coronavirus HKU15-MN3092(KJ584360)
Porcine coronavirus HKU15-MI6148(KJ620016)
Porcine coronavirus HKU15-KY4813(KJ584357)
Porcine coronavirus HKU15-IN2847(KJ569769)
Porcine coronavirus HKU15-IL2768(KJ584355)
Porcine coronavirus HKU15-HKU15-44(JQ065042)
Porcine coronavirus HKU15-NE3579(KJ584359)
Porcine coronavirus HKU15-HKU15-155(JQ065043)
Asian leopard cat coronavirus F230(EF584908)
Sparrow coronavirus HKU17-6124(JQ065045)
Thrush coronavirus HKU12-600(NC_011549)
Magpie-robin coronavirus HKU18-chu3(JQ065046)
Munia coronavirus HKU13-3514(NC_011550)
Bulbul coronavirus HKU11-796(FJ376620)
Night-heron coronavirus HKU19-6918(JQ065047)
Beluga Whale coronavirus SW1(NC_010646)
Infectious bronchitis virus YN(JF893452)
Turkey coronavirus(NC_010800)
Bat coronavirus HKU4-4(EF065508)
Tylonycteris bat coronavirus HKU4-1(NC_009019)
Pipistrellus bat coronavirus HKU5-1(NC_009020)
Bat SARS coronavirus HKU3-13(GQ153548)
SARS coronavirus TJF(AY654624)
Rousettus bat coronavirus HKU9-1(NC_009021)
Human coronavirus HKU1(NC_006577)
Murine hepatitis virus JHM(AC_000192)
Equine coronavirus NC99(NC_010327)
PHEV VW572(DQ011855)
Bovine coronavirus strain Mebus(BCU00735)
Human coronavirus OC43(NC_005147)
PRCV ISU-1(DQ811787)
TGEV-H16(FJ755618)
TGEV Miller M6(DQ811785)
TGEV virulent Purdue(DQ811789)
TGEV-PUR46-MAD(AJ271965)
Canine coronavirus NTU336/F/2008(GQ477367)
Feline coronavirus FIPV 79-1146(DQ010921)
Human coronavirus 229E J0304(JX503061)
Human coronavirus NL63 CBJ 037(JX104161)
Miniopterus bat coronavirus 1A(NC_010437)
Miniopterus bat coronavirus 1B(NC_010436)
Rhinolophus bat coronavirus HKU2(NC_009988)
PEDV-IA2(KF468A753)
PEDV-Colorado(KF272920)
PEDV-OH851(KJ399978)
PEDV-OH1414(KJ408801)
Deltacoronavirus
Gammacoronavirus
Betacoronavirus
Alphacoronavirus
0.1

Technical Appendix Figure. Phylogenetic tree constructed on the basis of the whole genome sequences (A), spike protein (B) and nucleocapsid protein (C) of 4 genera of coronaviruses (*Alphacoronavirus*, *Betacoronavirus*, *Gammacoronavirus*, and *Deltacoronavirus*), including the 9 US strains of porcine coronavirus HKU15 (indicated by red triangle). The dendrogram was constructed by using the neighbor-joining method in the MEGA software package, version 6.05 (http://www.megasoftware.net). Bootstrap resampling (1,000 replications) was performed, and bootstrap values are indicated for each node. Reference sequences obtained from GenBank are indicated by strain name and accession number. Scale bar represents 0.5 nt (A) or 0.1 aa (B, C) substitutions per site. PEDV, porcine epidemic diarrhea virus; PHEV, porcine hemagglutinating encephalomyelitis virus; PRCV, porcine respiratory coronavirus; TGEV, transmissible gastroenteritis coronavirus; SARS, severe acute respiratory syndrome.
